# Optical Coherence Tomography Angiography in the Thirteen-Lined Ground Squirrel

**DOI:** 10.1167/tvst.10.8.5

**Published:** 2021-07-07

**Authors:** Alexander E. Salmon, Rex Chin-Hao Chen, Farid Atry, Mina Gaffney, Dana K. Merriman, Daniel A. Gil, Melissa C. Skala, Ross Collery, Kenneth P. Allen, Eric Buckland, Ramin Pashaie, Joseph Carroll

**Affiliations:** 1 Cell Biology, Neurobiology, & Anatomy, Medical College of Wisconsin, Milwaukee, WI, USA; 2 Translational Imaging Innovations, Hickory, NC, USA; 3 Biomedical Engineering, University of Wisconsin-Madison, Madison, WI, USA; 4 Ophthalmology & Visual Sciences, Medical College of Wisconsin, Milwaukee, WI, USA; 5 Biology, University of Wisconsin-Oshkosh, Oshkosh, WI, USA; 6 Morgridge Institute for Research, Madison, WI, USA; 7 Microbiology & Immunology, Medical College of Wisconsin, Milwaukee, WI, USA; 8 Computer & Electrical Engineering, Florida Atlantic University, Boca Raton, FL, USA; 9 Biomedical Engineering, Marquette University, Milwaukee WI, USA

**Keywords:** optical coherence tomography-angiography, adaptive optics, retinal vasculature, correlative histology

## Abstract

**Purpose:**

To assess the performance of two spectral-domain optical coherence tomography-angiography systems in a natural model of hypoperfusion: the hibernating thirteen-lined ground squirrel (13-LGS).

**Methods:**

Using a high-speed (130 kHz) OCT-A system (HS-OCT-A) and a commercial OCT (36 kHz; Bioptigen Envisu; BE-OCT-A), we imaged the 13-LGS retina throughout its hibernation cycle. Custom software was used to extract the superior, middle, and deep capillary plexus (SCP, MCP, and DCP, respectively). The retinal vasculature was also imaged with adaptive optics scanning light ophthalmoscopy (AOSLO) during torpor to visualize individual blood cells. Finally, correlative histology with immunolabeled or DiI-stained vasculature was performed.

**Results:**

During euthermia, vessel density was similar between devices for the SCP and MCP (*P* = 0.88, 0.72, respectively), with a small difference in the DCP (−1.63 ± 1.54%, *P* = 0.036). Apparent capillary dropout was observed during torpor, but recovered after forced arousal, and this effect was exaggerated in high-speed OCT-A imaging. Based on cell flux measurements with AOSLO, increasing OCT-A scan duration by ∼1000× would avoid the apparent capillary dropout artifact. High correspondence between OCT-A (during euthermia) and histology enabled lateral scale calibration.

**Conclusions:**

While the HS-OCT-A system provides a more efficient workflow, the shorter interscan interval may render it more susceptible to the apparent capillary dropout artifact. Disambiguation between capillary dropout and transient ischemia can have important implications in the management of retinal disease and warrants additional diagnostics.

**Translational Relevance:**

The 13-LGS provides a natural model of hypoperfusion that may prove valuable in modeling the utility of OCT-A in human pathologies associated with altered blood flow.

## Introduction

Optical coherence tomography-angiography (OCT-A) enables depth-resolved, noninvasive assessment of the retinal and choroidal vasculature.^1^^–^^4^ This has had a profound impact on the clinical care and study of numerous pathologies, including retinal vein occlusion,^5^ diabetic retinopathy,^6^ age-related macular degeneration,^7^ sickle cell retinopathy,^8^ and choroideremia.^9^ Despite the numerous advantages of OCT-A over techniques such as fluorescein angiography,^10^ validation of the metrics extracted from OCT-A images with correlative histology are fairly sparse.^11^^,^^12^ In addition, OCT-A can be limited by several artifacts such as false positive and false negative flow signals caused by eye motion or the angiography algorithm.^13^ In particular, disambiguation between capillary dropout and transient ischemia may be especially challenging in OCT-A, as low blood cell flux induces similar levels of decorrelation as static tissue, resulting in limited dynamic contrast.^7^^,^^14^^,^^15^

The thirteen-lined ground squirrel (13-LGS; *Ictidomys tridecemlineatus*) offers a unique opportunity to test and validate some aspects of OCT-A imaging due to specialized physiologic features. The 13-LGS exhibits a holangiotic vascular system with major inner retinal vessels arranged in a parallel fashion exiting the horizontal optic nerve head (ONH), which in turn bisects the retina slightly superior to the posterior pole.^16^ As an obligate hibernator, the 13-LGS annually undergoes a state of metabolic suppression. During torpor (the state of hypometabolic heterothermy typically thought of as hibernation throughout the winter), body temperature may decrease to 2°C to 10°C, and heart rate decreases to 3 to 10 bpm (compared to 37°C and 200–300 bpm, respectively, in euthermia^17^^,^^18^). The ability to avoid cell death and organ failure in states of hypothermia, hypoxia, hypercapnia, and ischemia are of general translational interest, but the reduced heart rate and accompanying decrease in blood flow creates an opportunity to probe the limits of OCT-A hardware and software parameters to assess the retinal vasculature.

To this end, we imaged the 13-LGS retina throughout its hibernation cycle using a custom high-speed (130 kHz), spectral-domain OCT-A system, as well as a commercial OCT system with modified processing software. In the torpid state, the lack of perfusion in small capillaries manifested as apparent capillary dropout, an artifact which partially recovered after the animals approached normothermia. Vessel density measurements were compared between devices to assess reproducibility, as this is likely to be a useful biomarker in potential disease models generated in the 13-LGS. Finally, correspondence between in vivo OCT-A images and ex vivo images of the retinal vasculature after cardiac perfusion with a lipophilic dye was assessed,^19^ which enabled an empirical calibration of lateral image scale and comparison to an optical model of the 13-LGS eye.

## Methods

### Custom High-Speed OCT-A System Hardware

**Figure 1.**
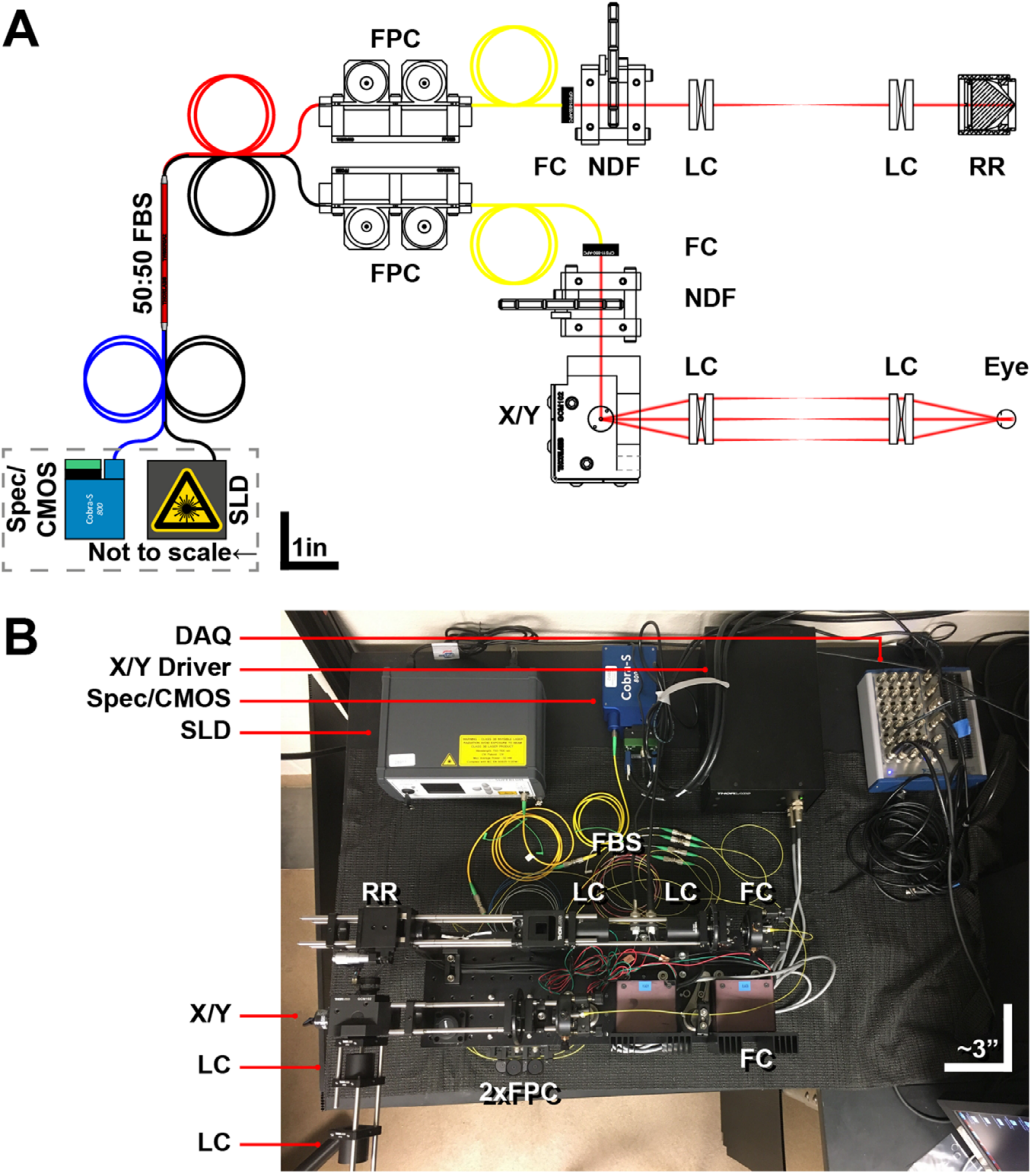
Custom HS-OCT-A hardware. (**A**) Schematic and (**B**) photograph of the custom HS-OCT-A system. Theoretical axial and lateral resolution: 1.5 and 2.0 µm, respectively. SLD: Superluminescent diode (Superlum; 850/165 nm; ∼3.25 mW); Spec/CMOS: spectrometer/camera (Wasatch; 840/180 nm; 130 kHz); FBS: fiber beam splitter; FPC: fiber polarization controller; FC: fiber collimator; NDF: neutral density filter wheel; X/Y: galvonometer; LC: lens compound; RR: retroreflector; DAQ: data acquisition device.

The overall design of the custom high-speed OCT-A device, henceforth referred to as HS-OCT-A, is a fiber-based spectral-domain OCT (Fig. 1). A broadband superluminescent diode (SLD; M-T-850-HP-I, Superlum, Carrigtwohill, Co. Cork, Ireland) was selected with a center wavelength (λ_0_) of 850 nm and a bandwidth (Δλ) of 165 nm. This light source was chosen to improve light safety,^20^^,^^21^ avoid absorption by ocular media,^22^^,^^23^ and obtain a theoretical axial resolution of 1.5 µm in tissue^24^ to improve detection of the smallest capillaries (estimated to be at least 3.2 µm in diameter in mice).^14^^,^^25^ The light is split by a wideband 50:50 fiber coupler (TW850R5A2; Thorlabs, Newton, NJ) into the sample and reference arms, where it is collimated into 2.1 mm beams by pigtailed aspheric fiber collimators (CFS11-850-APC; Thorlabs).

The sample arm contains a 2-paddle fiber polarization controller (FPC; FPC023; Thorlabs), a neutral density (ND) filter wheel (NDM2; Thorlabs), a dual-axis galvanometer system with gold-coated mirrors (GVS102; Thorlabs), and a Plössl lens configuration (two pairs of achromatic doublets, each with a focal length of 100 mm; AC254-100-B; Thorlabs). These lenses were selected to achieve an effective focal length of 51.05 mm (with a separation of ∼4 mm between lens pairs) and to minimize astigmatism and field curvature.^26^ A working distance of at least 50 mm is desirable when imaging the 13-LGS and other species to avoid positioning interference with the rotational stage and anesthesia equipment.^27^ With these hardware parameters, we calculate a theoretical diffraction-limited lateral resolution of ∼2.0 µm in the 13-LGS eye^28^^–^^31^ (Appendix), although ocular aberrations are uncorrected, so this is certainly an overestimate, and current eye models for the 13-LGS are not as well developed as for humans and mice. Optical power at the cornea was measured to be 0.05 to 3.25 mW depending on the ND filter wheel position using a power meter (1931-C; Newport Corporation, Irvine, CA) set to the center wavelength; the maximum power was used for all images included in this study, as this was calculated to be safe for all scan protocols used (Appendix). The reference arm also contains an FPC, NDM2, and a set of AC254-100-B lenses to match the sample arm. A retroreflecting prism (PS975M-B; Thorlabs) mounted on a cage-compatible translation stage (CT1; Thorlabs) at the end of the reference arm was used to adjust for variations in eye axial length.

Light collected by the sample and reference arms is then sent to a commercial spectrometer assembly, which terminates in a 1 × 2048 pixel complementary metal oxide semiconductor (CMOS) camera (CS800-840/180-250-OC2K; Wasatch Photonics, Morrisville, NC), which was selected to closely match the light source and lower the noise floor. The spectrometer has a wavelength range of 750 to 930 nm (light source: 767.5–932.5 nm) and a maximum depth range (in air) of 2.0 mm. In the current configuration, the CMOS camera has a maximum A-scan rate of 130 kHz (129.5 kHz used for this study to avoid synchronization errors) at 12 bits/pixel. Frames are digitized by a frame grabber (PCIe-1433, National Instruments, Austin, TX), which was synchronized to the galvanometers with a data acquisition device (DAQ; USB-6363, National Instruments). Hardware and software were controlled with a custom PC with an AMD 8-core CPU (4.0 GHz), 32GB RAM, and an NVIDIA Quadro K620 GPU.

### Acquisition and Post-Processing Software for the High-Speed OCT-A System

**Figure 2.**
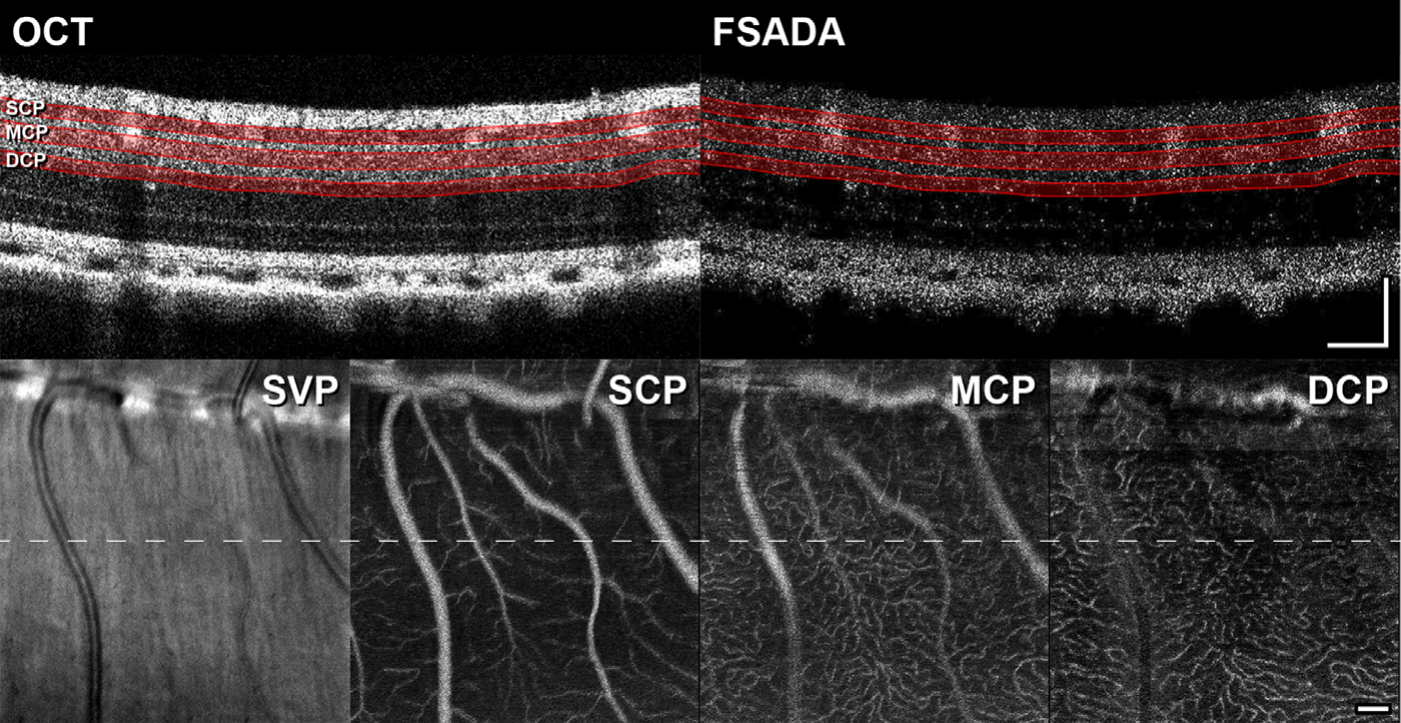
BM-scans and *en face* images in the 13-LGS. A registered, averaged, log-scaled structural BM-scan (four repeated B-scans at the same retinal location; OCT) from animal 165310 acquired with the HS-OCT-A. All outer-retinal hyperreflective bands are visible, which demonstrates the multipurpose utility of this device for high-resolution structural and functional imaging. (**B**) Full-spectrum amplitude decorrelation angiography (FSADA) image generated from the same BM-scan. Representative segmentations are shown on both the structural OCT and FSADA images for the superior, middle, and deep capillary plexuses (SCP, MCP, and DCP, respectively). False negative flow signal from the choroid and false positive flow signal from the surrounding nonvascular tissue is a consequence of the structural amplitude-based masking employed in our implementation of the FSADA algorithm; the choroidal vasculature was not analyzed in this study. The summed volume projection (SVP) from the structural OCT as well as the summed volume projection from the SCP, MCP, and DCP are shown; *dashed line*: location of the BM-scan. Scale bars: 100 µm.

Acquisition software for the custom device was developed in LabVIEW 2017 (National Instruments), requires NI-IMAQ and NI-Vision toolboxes, and was adapted from software used previously for OCT-A in the mouse brain.^32^ Real-time OCT image processing (including DC-term and autocorrelation mitigation, *k*-space interpolation, dispersion compensation, and Fourier transformation) was implemented in LabVIEW using built-in parallel processing architecture to enable video-rate display. Conversion to *k*-space was achieved according to the manufacturer's instructions (Wasatch Photonics) with spline interpolation. DC-term and autocorrelation mitigation was performed by subtracting the mean spectral intensity across line scans in each frame from each line scan. Dispersion compensation was achieved by multiplying each line scan by a phasor,^33^ with coefficients determined by a custom nonlinear optimization approach (Appendix). The amplitude of the real part of the Fourier transform was then log transformed for display to the user (Appendix). The acquisition software allows for arbitrary fields of view, sampling, scan rotation, as well as BM- (repeated B-scanning at a given location) and CM-scanning (repeated volume scanning); specific scan parameters used in this study are listed in *Anesthesia, OCT-A Imaging*. It also allows for data acquisition either only on the forward scan or on both the forward and reverse scan, with automatic reflection and averaging; although only forward scanning was used in this study, as this simplifies calculation of the interscan intervals used in BM-scanning. The output of the software is a binary file containing only the raw camera images, a separate header file (.txt) with all the scan parameters required for reading in a separate application, as well as an *en face* mean intensity projection from structural and angiographic volumes for image quality feedback. For these feedback images, no frame registration within a BM-scan was conducted (to increase speed) but was conducted offline using a custom nonlinear optimization of translation and vertical shear (Appendix, Supplementary Fig. S1). The angiography algorithm used was full-spectrum amplitude decorrelation angiography (FSADA; Fig. 2).^34^ Structural volumes were segmented using a custom platform, and the segmentations were applied to the angiographic volume to obtain *en face* images for longitudinal alignment and ROI selection (Appendix).

### Commercial OCT System Hardware and Software

We also developed open-source software^35^ to generate angiograms using a Bioptigen Envisu R2200 OCT device (Leica Microsystems, Wetzlar, Germany), henceforth referred to as BE-OCT-A. This device is equipped with a custom light source (Superlum Broadlighter T870; λ_0_: 878.4 nm, Δλ: 186.3 nm; Superlum) and has a theoretical axial resolution of 1.4 µm. Bioptigen's Gen 3 “rabbit bore” was used for retinal imaging, which allows for fine focus adjustment and has a reported beam diameter of ∼1.7 mm at the cornea, resulting in a theoretical lateral resolution of 2.4 µm (Appendix). When a scan was acquired, the .OCU file (which contains the raw camera images) was output to enable offline processing of unaltered data. Key parameters required for *k*-space interpolation are available in Bioptigen .INI files, so these are required inputs to the OCT-A processing pipeline. We adapted a custom MATLAB (MathWorks, Natick, MA) function provided by Bioptigen to read individual frames from the .OCU file (so as not to overload the RAM by reading the entire volume), at which point the images could be processed with the same functions as the custom device.

### Animals and Thermic State Definitions

The experimental procedures described were approved by the Institutional Animal Care and Use Committee of the Medical College of Wisconsin (MCW), which is fully accredited by AAALAC International, and were in accordance with the ARVO Statement for the Use of Animals in Ophthalmic and Vision Research. All animals were obtained from the University of Wisconsin-Oshkosh Squirrel Colony for use in this study at MCW. Nonhibernating animals were housed at room temperature with a natural photoperiod, with light adjusted every two weeks to mimic that of southern Wisconsin. Animals were housed in static micro isolation cages (Allentown 140, Allentown Caging, Allentown, NJ) The environment in the rooms housing animals was controlled (68°F to 72°F [20.0°C to 22.2°C]; relative humidity, 30% to 70%). Three cohorts of animals were included in this study based on thermic states. One cohort was imaged only in the euthermic state. The second cohort was allowed to hibernate and examined during torpor, then warmed to nearly normal body temperature and re-examined, henceforth referred to as torpid/warmed (T/W). The final cohort was imaged during euthermia and during the winter months but was prevented from hibernating; since significant deviations in physiology have been noted immediately preceding and during winter months.^17^^,^^36^ This cohort represents a pseudo-euthermic physiology and is referred to as winter-active (WA:Eu). A summary of animal groupings is given in Table.

**Table.** Animal Demographics, Thermic States, Imaging Modalities, and Endpoints

ID	Sex	Ages (Y)	Cohort	HS & BE	AOSLO	Endpoint
164107	M	3.9–4.0	Eu	+		α-SMA IHC
165310	F	3.9–4.0	Eu	+		DiI CP
164202	F	4.3	Eu	+		DiI CP
180402	F	2.3–2.4	Eu	+		DiI CP
187903	M	1.6–2.0	WA:Eu	+		α-SMA IHC
175003	M	2.5–3.3	WA:Eu	+		DiI CP
154802	F	4.4–5.3	WA:Eu	+		DiI CP
186302	F	1.5–1.8	T/W	+	+	NA
164807	M	3.5–4.3	T/W	+		DiI CP
164904	F	3.5–4.3	T/W	+		DiI CP
165308	F	4.1	N/A			DiI CP

Abbreviations: HS & BE: high-speed and Bioptigen Envisu OCT-A; AOSLO: adaptive optics scanning light ophthalmoscopy; Eu: euthermic; WA: winter-active; T/W: torpid/warmed; α-SMA IHC: α-smooth muscle actin immunohistochemistry; DiI CP: 1,1’-dioctadecyl-3,3,3’,3’-tetramethylindocarbocyanine perchlorate cardiac perfusion. Animal 186302 died unexpectedly after hibernation so histology could not be collected. Animal 165308 was used only to optimize DiI CP parameters.

### Hibernation Monitoring and Timeline of Study

The T/W cohort was placed in a dark 4°C hibernaculum (True Manufacturing, O'Fallon, MO) without food or water and allowed to hibernate for over one month before they were used for this study. Animals in the hibernaculum were checked daily for activity (cage condensation, motion, or vocalizations), which indicates either a period of interbout euthermia (a normal cycle of activity that lasts approximately eight hours) or a failure to hibernate. Animals which were found to be active for three consecutive days would be moved back to room temperature and lighting for the remainder of the season. Hibernation failure occurred in one animal (175003), which became part of the winter-active cohort, but it was only examined more than one month after transition to room temperature. The T/W cohort was examined between November 2019 and March 2020. The WA:Eu cohort was examined during the same period as T/W, then re-examined between April and August 2020. The euthermic-only cohort was only examined between April and August 2020.

### Anesthesia, OCT-A Imaging

Euthermic animals were anesthetized with inhaled isoflurane (3%–5% for induction in a chamber, 1%–4% maintenance via mask delivery) with 1 L/min O_2_ flow using a nonrebreathing system (VetEquip, Inc., Livermore, CA). Dilation and cycloplegia were achieved with one drop each of 2.5% phenylephrine hydrochloride and 1% tropicamide (Akorn, Inc., Lake Forest, IL). Whiskers were matted down with Vaseline, and the eyes were held open with a pediatric ocular speculum. Wetting drops (Refresh Drops; Allergan Pharmaceuticals, Dublin, Ireland) were applied as needed to maintain corneal hydration and an even tear film. An animal was placed on a stage with five degrees of freedom (X, Y, Z, roll (superior-inferior retinal axis), and yaw (nasal-temporal retinal axis)), which allowed alignment of the pupil, matching the optical path length of the reference arm, and steering to the appropriate retinal location. For the HS-OCT-A, isotropic scans with 7°, 10°, and 12° FOVs were acquired with 467, 667, and 800 A-scans/B-scans and B-scans/volume, respectively, with between two to eight repeated B-scans per location. Repetition time (T_R_; the delay between imaging the same location in a BM-scan) ranged between 5.03 to 8.42 ms. For the BE-OCT-A, all scans had a nominal scan size of 5 × 5 mm with 450 A-scans/B-scan, 450 B-scans/volume, with four repeated B-scans per location. By scaling *en face* images from the BE-OCT-A to the HS-OCT-A, it was determined that the approximate lateral pixel scale was 2× larger than that of the HS-OCT-A (i.e., a 5 mm nominal scan length for the BE-OCT-A corresponds to ∼13.5° for the HS-OCT-A in the 13-LGS). T_R_ for the BE-OCT-A was approximately 13 ms.

### Special Conditions for OCT-A Imaging of the Torpid/Warmed Cohort

Animals in the T/W cohort were transported between the hibernaculum and the examination room while inside an insulated foam cooler to minimize light, sound, and heat exposure. All imaging was conducted with the room lights off and computer monitors dimmed; additional procedural illumination was provided by a dim red headlamp. Body temperature was measured remotely using a thermal imaging camera (FLIR E60; FLIR Systems, Inc., Wilsonville, OR). A flexible cooling pack wrapped in paper towel was applied to the animal's back to maintain an external temperature similar to that of the hibernaculum; a new cooling pack was applied approximately once per hour during the imaging session. Anesthesia induction was not required, but isoflurane was maintained between 1% to 2% during imaging. Dilation, cycloplegia, and lid opening were conducted as above. Once images were acquired from an animal in a torpid state, the room lights were turned on, the cooling packs were removed, and the animal was moved to a cage resting on a thermal heat pack for approximately two hours. Once the animal was capable of ambulation and the thermal imaging camera indicated a body temperature of ≥ 26.5°C, anesthesia was induced (3%–4% isoflurane) and dilation and cycloplegia were repeated. During the “warmed” segment of the imaging session, a thermal heat pack was applied to the underside of the PVC imaging stage to reduce the risk of re-entrance into torpor.

### Vessel Segmentation and Quantification

Vessel density was quantified using a custom algorithm implemented in MATLAB. A single nominal scale of 1.5 µm/pixel was used to calibrate segmentation parameters. *En face* angiograms were first passed through a Gaussian filter (σ = 3 µm) followed by a Hessian-based Frangi vesselness filter (σ = 4–10 pixels, 1-pixel steps) to produce maximum vesselness projection images (each pixel represents the maximum vesselness across all scales).^37^^,^^38^ The filtered images then underwent adaptive local thresholding^39^ with a sensitivity of 1_E_^−6^ and a window side length of 50 µm. The binarized images were then refined by removing floating segments with total areas less than 81 µm^2^ (36 pixels) to mitigate false-positive signals originating from noise. Vessel density was expressed as a percentage of positive pixels over the total number of pixels in the ROI. Additionally, agreement between devices was assessed by computing the Dice coefficient (*Dice*  =  (2(*HS*∩*BE*))/(*HS* + *BE*)) for binarized images pairs.

### Adaptive Optics Imaging and Image Processing

To examine individual blood cell flux, one animal (186302) was imaged during torpor with a previously described adaptive optics scanning light ophthalmoscope (AOSLO).^40^ We focused to the MCP and collected confocal reflectance videos of the capillaries using 790 nm light and a frame rate of 16.6 Hz. Using a 30 µm pinhole (0.7 Airy disk diameter), confocality was sufficient to reject light from the adjacent vascular layers and the nerve fiber layer, though the axial resolution has not yet been empirically determined for the 13-LGS with this system.^41^ Images of a Ronchi ruling with known spacing were acquired to correct for the static sinusoidal distortion induced by the resonant scanner in this system and to calibrate lateral image scale. A minimally distorted reference frame was automatically selected,^42^ and the remaining frames in the video were subjected to full-frame registration by normalized cross-correlation.^43^ From the registered video, the positions of a pair of individual blood cells (presumably erythrocytes based on size) was tracked and their motion calculated based on frame time stamps.

### Immunohistochemical Labeling of Retinal Vasculature

To assess the retinal vasculature without the confounds of eye motion and the limiting optics of the eye, we performed location-matched immunohistochemical labeling of anti-α-smooth muscle actin (α-SMA). Animals 164107 and 187903 were deeply anesthetized with 5% isoflurane in 1 L/min O_2_ until breathing was substantially reduced. Animals were euthanized by decapitation, the eyes were removed, and stored overnight in 4% paraformaldehyde (PFA) in 1x PBS, pH 7.4. Each eye was rinsed and submerged in PBS three times for five minutes at room temperature (RT). The anterior segment was removed, and the posterior segment tissue was submerged in blocking buffer (2% normal donkey serum in PBS-T: 1% Triton X-100/1% Tween-20 in PBS) for one to six hours at RT. The tissue was then incubated with rabbit polyclonal anti-α-smooth muscle actin (α-SMA; 1:1000 in blocking buffer; Abcam 15734) for three to four days with gentle rotation at RT. Tissues were rinsed briefly, then washed with rotation 3 × 60 minutes in PBS-T (pH 7.4) at RT. Tissues were then incubated with donkey anti-rabbit-Alexa Fluor 488 (1:750 in blocking buffer, Life Tech A21206) in the dark, overnight at 4°C. The tissues were then washed 4 × 30 minutes in PBS-T. Circumferential cuts (four to eight) were made in the posterior segment, and the sclera was peeled away. The retina was flat-mounted in cold 1:1 glycerol/PBS on a microscope slide, and the coverslip was sealed with clear nail polish. The sample was imaged with a Nikon Eclipse-80i confocal microscope (Nikon Corporation, Tokyo, Japan), using a 10× objective, with 488 nm illumination and a 525 nm fluorescence filter. Once the region previously imaged with OCT-A was identified, the *en face* angiograms were used to identify a best focus for each layer, then a 1024 × 1024 pixel (1.275 × 1.275 mm) image with 16× frame averaging was acquired. Images were scaled from 12-bit to 16-bit intensities and output as a .tif file, then manually aligned to the OCT-A images in Adobe Photoshop CS6 (Adobe Inc., San Jose, CA).

### Cardiac Perfusion Labeling of Retinal Vasculature

We performed cardiac perfusion with 1,1’-dioctadecyl-3,3,3’,3’-tetramethylindocarbocyanine perchlorate (DiI) using a slightly modified protocol.^19^ The animals were deeply anesthetized with 5% isoflurane in 1 L/min O_2_, and then a pneumothorax was induced. After cessation of breathing, but while the heart was still beating, the descending aorta was clamped with a hemostat, the right atrium was severed, and a 23G butterfly needle was inserted into the left ventricle. Connected to the butterfly needle was a system of three 10 mL syringes, each containing 1xPBS, pH 7.4, DiI solution (∼0.1 mg/mL final concentration in 1:4 1xPBS and 5% glucose), or 4% PFA in 1xPBS, controlled by 3-way stopcocks. The system of syringes was placed in a syringe pump set to 1.5 mL/min, and 2 mL PBS, 10 mL DiI, and 10 mL PFA were sequentially perfused into the heart. Immediately following perfusion, the animal was decapitated, the eyes were carefully removed, then stored in 4% PFA on ice until ready for dissection.

The optic nerve, extraocular muscle tissue, and extraorbital fat were trimmed from the back of the sclera. The anterior segment was removed, and the posterior segment was whole-mounted (sclera and RPE still attached) onto a cavity slide (diameter: 15–18 mm, 600–800 µm depth; Sigma: BR475505-50EA, BRAND GmbH + Co KG, Wertheim, Germany) in a cold 1:1 glycerol/PBS solution. A coverslip was then affixed to the cavity slide using clear nail polish. The sample was imaged with a Nikon Eclipse-80i confocal microscope (Nikon Corporation), using a 10× objective, with 561 nm illumination, and a 595 nm fluorescence filter. Once the region previously imaged with OCT-A was identified, a 512 × 512 pixel (1.275 × 1.275 mm) z-stack with ∼1 µm spacing over a range of ∼100 µm was acquired, with 4× frame averaging at each step. Images were scaled from 12-bit to 16-bit intensities and output as a .tif series or .nd2 stack, where they were further analyzed in ImageJ.^44^ Using the OCT-A *en face* images as a guide, the DiI micrographs were examined to identify a z-plane with matching features. Occasionally, the sample would be slightly tilted and not all vessels would be visible in the same plane; in these cases, a maximum intensity projection was computed over a small depth range (∼5 µm). DiI images were manually aligned to OCT-A images in Photoshop (translation, rotation, and scale), and then the OCT-A images were warped to the DiI images using bUnwarpJ^45^ to assess correspondence.

## Results

### Vessel Density Assessment Between OCT-A Devices

**Figure 3.**
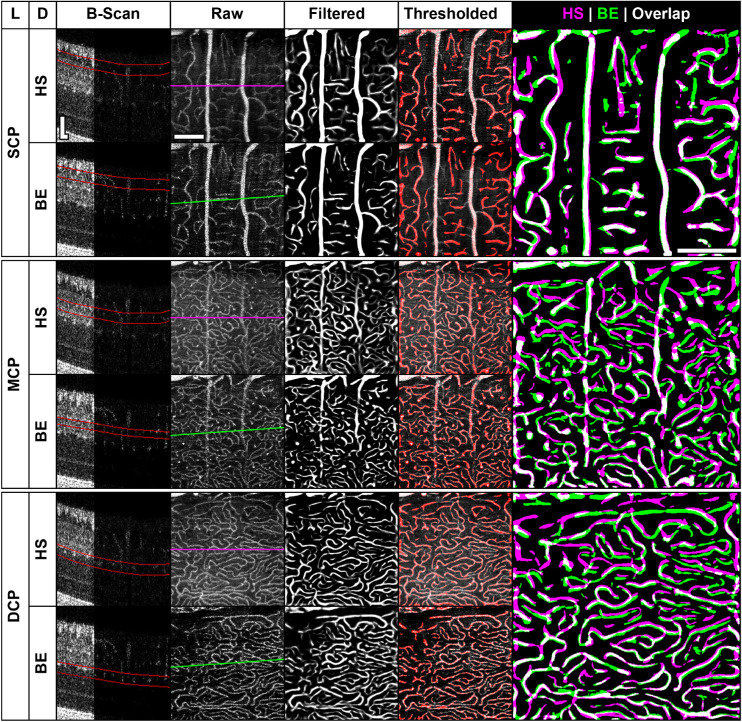
Vessel segmentation comparison for each OCT-A device. Superior, middle, and deep capillary plexus (SCP, MCP, and DCP, respectively) images acquired during euthermia in a representative animal (175003) are shown for the high-speed (HS) and Bioptigen Envisu (BE) OCT-A devices. Structural and angiographic B-scans (first 1/3 and last 2/3 of panel, respectively) from the location indicated by the lines on the *en face* angiograms (*magenta*: HS, *green*: BE) are shown with their corresponding segmentations outlined in red. Filtered images have been manually contrast stretched for display purposes. While the filtered images were thresholded for the vessel density computation, the thresholded images are overlaid on the raw images to facilitate identification of false positive and negative segmentations. This image set was not subjected to elastic registration to illustrate the magnitude of local distortions. B-scan scale bars: 50 µm; *en face* scale bars: 200 µm. Abbreviations: L: layer; D: device.

**Figure 4.**
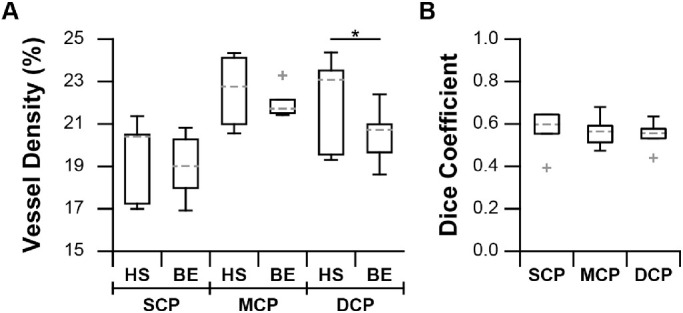
Comparison of vessel detection between devices. (**A**) Vessel density measurements from the same retinal location were compared between devices for each capillary plexus in seven euthermic animals. Differences between devices for a given layer were assessed by *n*-way ANOVA with post hoc correction (*P < 0.05). Boxes: interquartile range (IQR; 25^th^ to 75^th^ percentile); dashed line: median; whiskers: limits of observations excluding outliers (+) defined as an observation 1.5*IQR away from the 25^th^ or 75^th^ percentile. Abbreviations: BE: Bioptigen Envisu; HS: high-speed; SCP, MCP, DCP: superior, middle, and deep capillary plexus, respectively. (**B**) Dice coefficients between devices for thresholded images (1: perfect overlap; 0: no overlap). Overall, relatively good correspondence was found between devices, though errors in segmentation, filtration, registration, and binarization all reduce the Dice coefficient.

Correspondence in vessel detection between devices was assessed under typical imaging conditions. From location-matched ROIs (area: 0.6 mm^2^) acquired with both the HS- and BE-OCT-A from 13-LGS during euthermia (*n* = 7), vessels were segmented (Fig. 3), and vessel density and the Dice coefficient was compared between devices (Fig. 4). For this dataset, all BE-OCT-A images were acquired with four frames/B-scan, six out of seven HS-OCT-A images with two frames/B-scan (including animal 175003; Fig. 3), and one out of seven HS-OCT-A images with three frames/B-scan. Differences in vessel density were analyzed using an *n*-way ANOVA with a Tukey-Kramer post hoc test (Fig. 4A). While there was a significant difference in density between devices overall (*P* = 0.0051), the interaction between device and layer was not significant (P = 0.18). There was not a significant difference in density between devices for the SCP or MCP (*P* = 0.88, 0.72, respectively), and there was a small but significant difference for the DCP (*P* = 0.036, estimated difference: −1.63 ± 1.54%; µ ± 95% CI), suggesting that either the BE- or HS-OCT-A slightly underestimated or overestimated vessel density in the DCP, respectively. Median Dice coefficients were 0.60, 0.56, and 0.56 for the SCP, MCP, and DCP, respectively (Fig. 4B). Together, these results suggest that under these conditions (which represent the most common use case for imaging in this species) the custom HS-OCT-A device exhibits similar performance to that of the commercial BE-OCT-A device but includes a ∼3.6× speed improvement.

### Apparent Capillary Dropout Artifacts During Torpor

**Figure 5.**
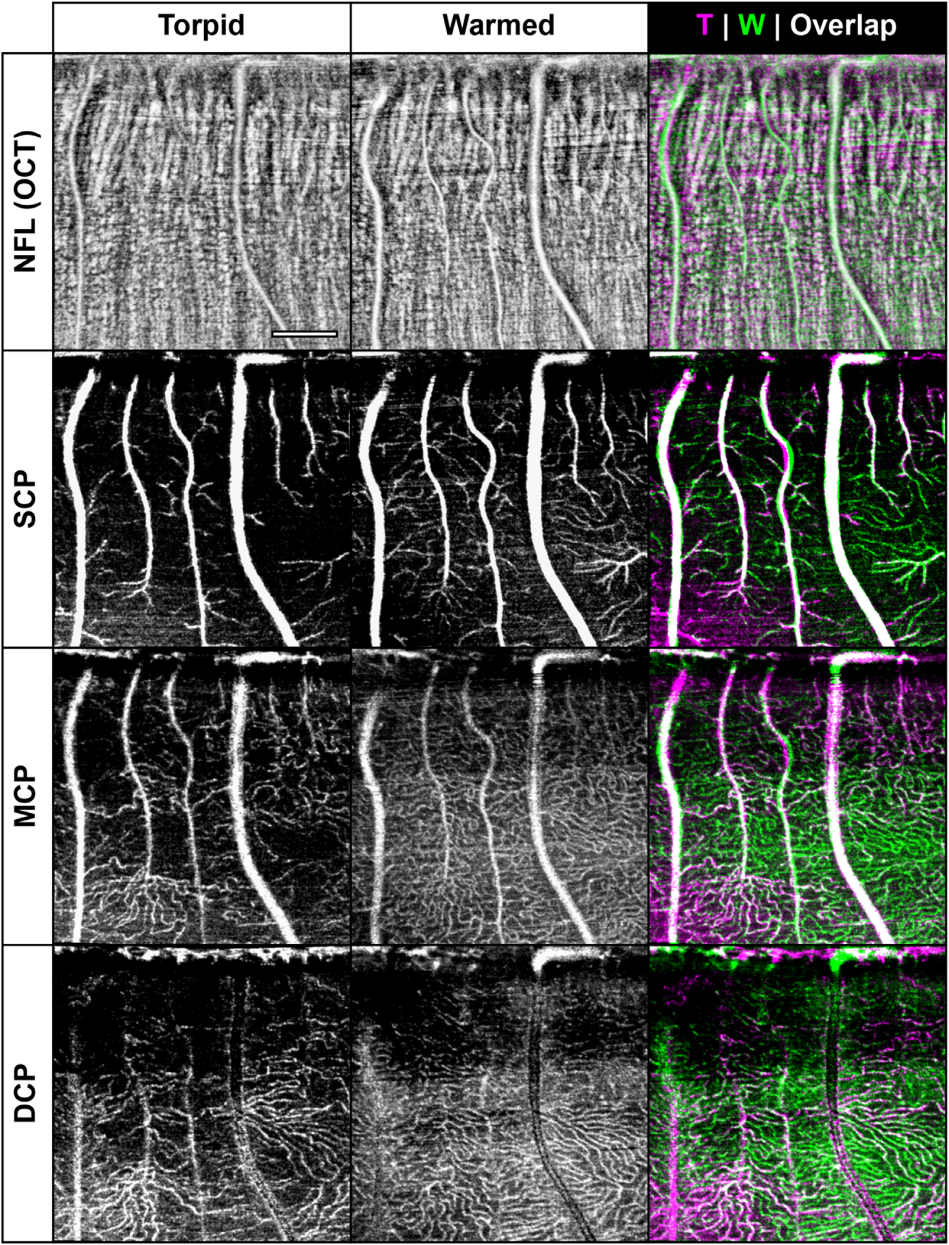
Apparent capillary dropout artifact during torpor. Animal 164807 was imaged with the Bioptigen system during torpor, wherein many capillaries appear absent, an effect which partially recovers after forced arousal (warmed). The large vessels shown in this region appear relatively unaffected by thermic state, and there does not appear to be a correlation with distance from the optic nerve head (ONH; top of ROI), suggesting a more complicated cause than simple diffusion. This animal was chosen for display due to the large overlapping area between thermic states, but all animals exhibited this effect (see Fig. 6). Scale bar: 300 µm. Abbreviations: NFL: nerve fiber layer; SCP, MCP, DCP: superior, middle, and deep capillary plexus, respectively; T: torpid; W: warmed.

**Figure 6.**
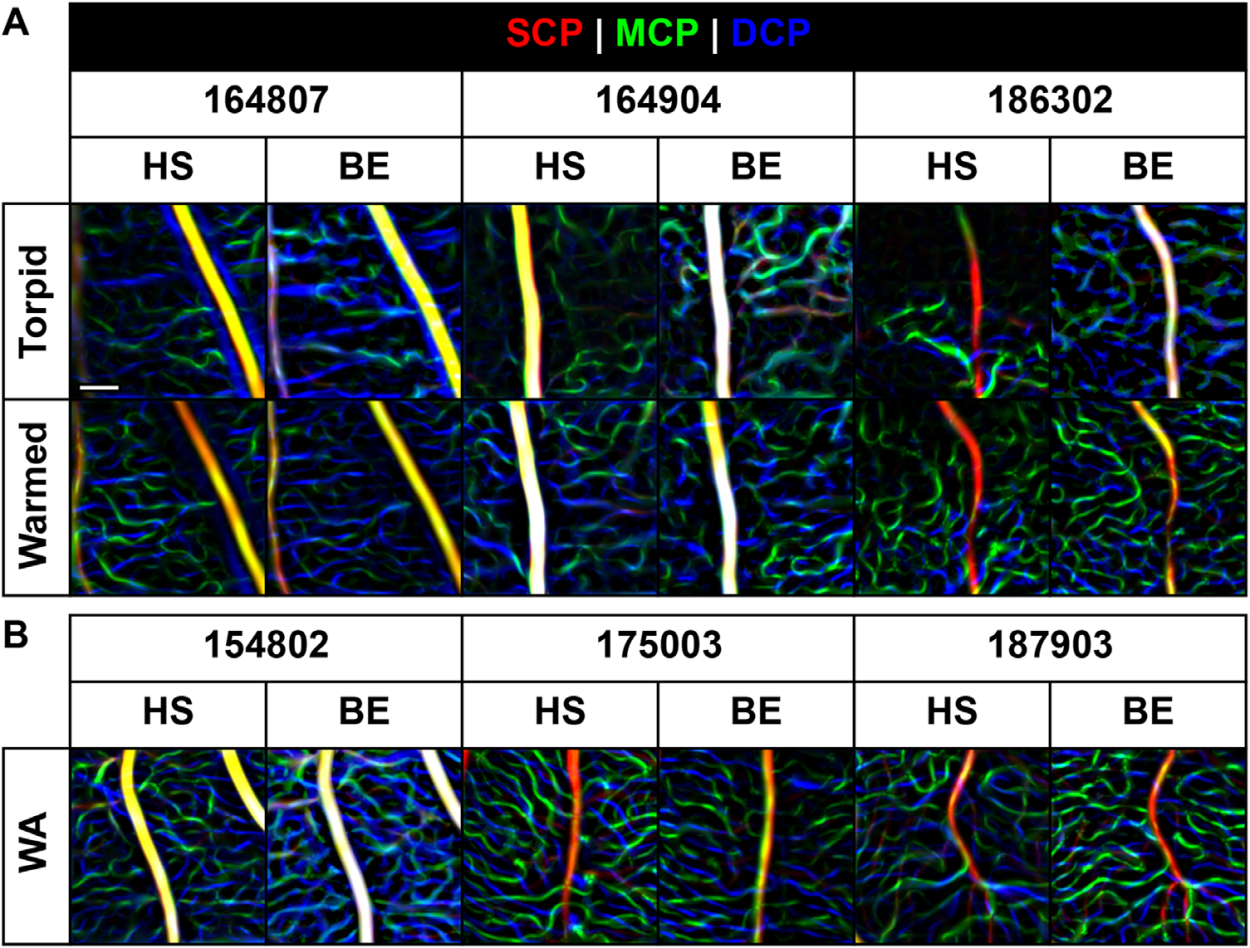
Comparison between devices for torpid, warmed, and winter-active animals. (**A**) Shown are location-matched, depth-encoded images of the vasculature during torpor and after forced arousal. Regions of apparent nonperfusion are especially prevalent in the high-speed (HS) OCT-A images compared to the Bioptigen Envisu (BE). Low blood cell flux combined with a shorter interscan interval may explain the disparity between devices in this state. (**B**) The similarity between images from a separate cohort of winter-active animals provides a frame of reference for the relatively small amount of variation due to scan quality and image processing. Further, it suggests that the cause of the apparent capillary dropout is related to the state of torpor as opposed to the season. The brightness of each image was globally adjusted for display purposes. Scale bar: 100 µm.

**Figure 7.**
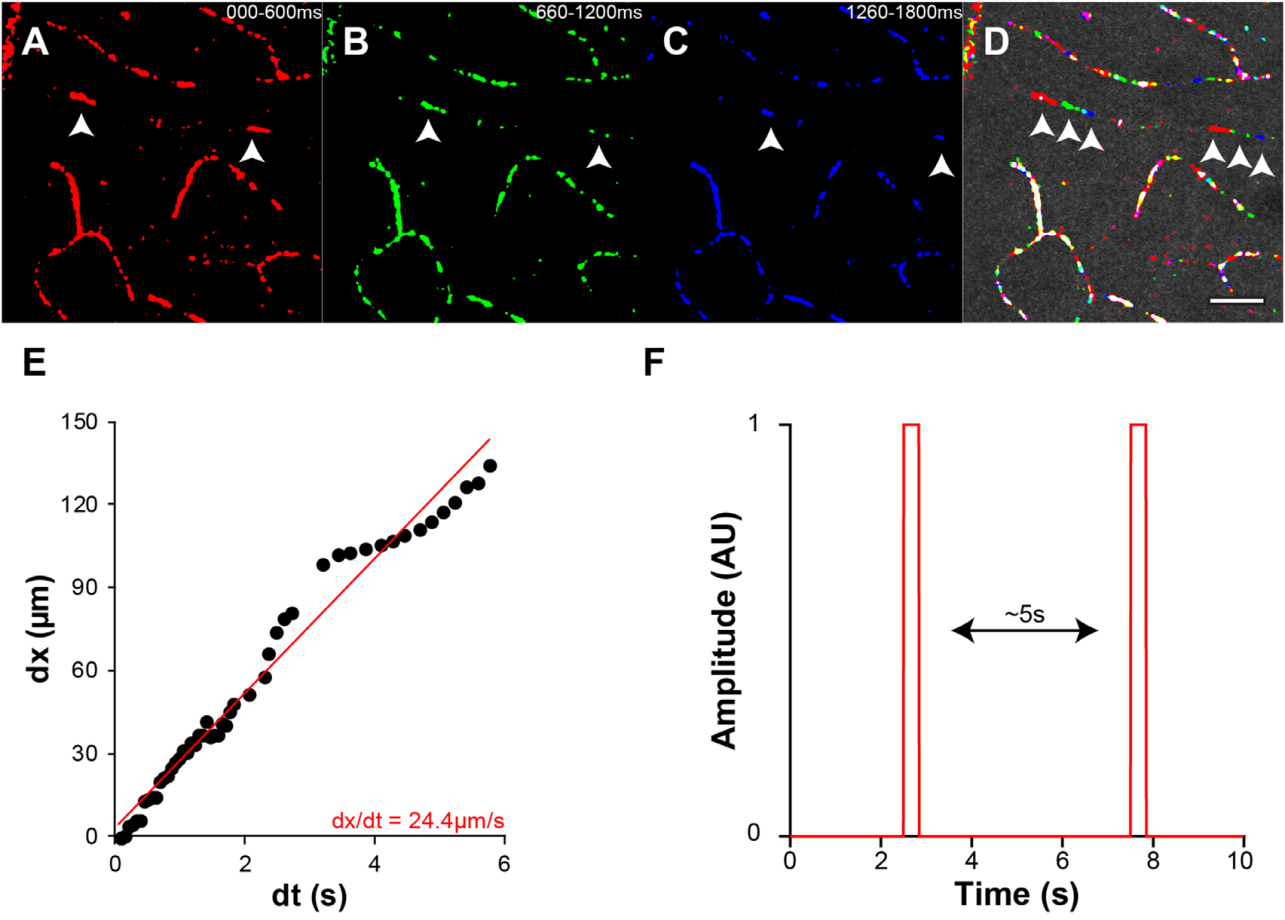
Single blood cell velocity during torpor. The middle capillary plexus was imaged in animal 186302 during torpor with AOSLO. (**A**–**C**) Standard deviation images from subsequent 10-frame bins, manually binarized. Arrow heads indicate a pair of blood cells within a single capillary used for velocity measurement. (**D**) Color merge overlaid on one frame of the raw reflectance image. See also Supplementary Video 1. Scale bar: 35 µm. (**E**) The positions and sizes of the pair of blood cells indicated in (**D**) were measured to obtain estimates of velocity and duty cycle to generate a square wave model (**F**). We estimate that each retinal location would need to be scanned for approximately five seconds to obtain an OCT-A *en face* image wherein a fully connected capillary could be detected (∼1000× longer than current OCT-A interscan intervals). This is impractical and would most likely suffer from prominent eye-motion artifacts.

To assess the performance of the OCT-A devices in a state of altered blood flow, we took advantage of the natural hibernation cycle in the 13-LGS. Using both OCT-A devices, we imaged a subset of the animals during torpor and then reimaged after forced arousal (T/W cohort; Table). In several locations, the retinal vasculature appeared quite sparse during torpor, an effect which partially recovered after being warmed to nearly normal body temperature (Fig. 5). This effect was seen in capillaries at all vascular plexuses but was apparently not strong enough to ablate the signal in large and medium caliber vessels of the SCP, whose detection persisted in the torpid and warmed state. When comparing between devices there appeared to be a predilection for the HS-OCT-A to detect fewer vessels during torpor than the BE-OCT-A (Fig. 6). To further investigate this effect, we imaged the MCP of one animal (186302) during torpor with AOSLO (Fig. 7; Supplementary Video 1). Motion-stabilized confocal reflectance videos were used to visualize individual blood cell flux through small capillaries. Adjacent capillaries could be seen with highly variable flow rates, which did not appear to correlate with distance from the ONH. This suggests that the low flux in certain vessels is not completely explained by low pressure from the supply source. We tracked the positions and sizes of a pair of blood cells in one video to measure velocity and approximate duty cycle of a square wave model of OCT-A contrast over time. The velocity of this pair of blood cells was 24.4 µm/s on average, each with a length of 8.4 µm, and a separation of 122 µm, yielding a square wave duty cycle of only 7%.

### OCT-A to Histological Comparison and Multimodal Lateral Scale Calibration

**Figure 8.**
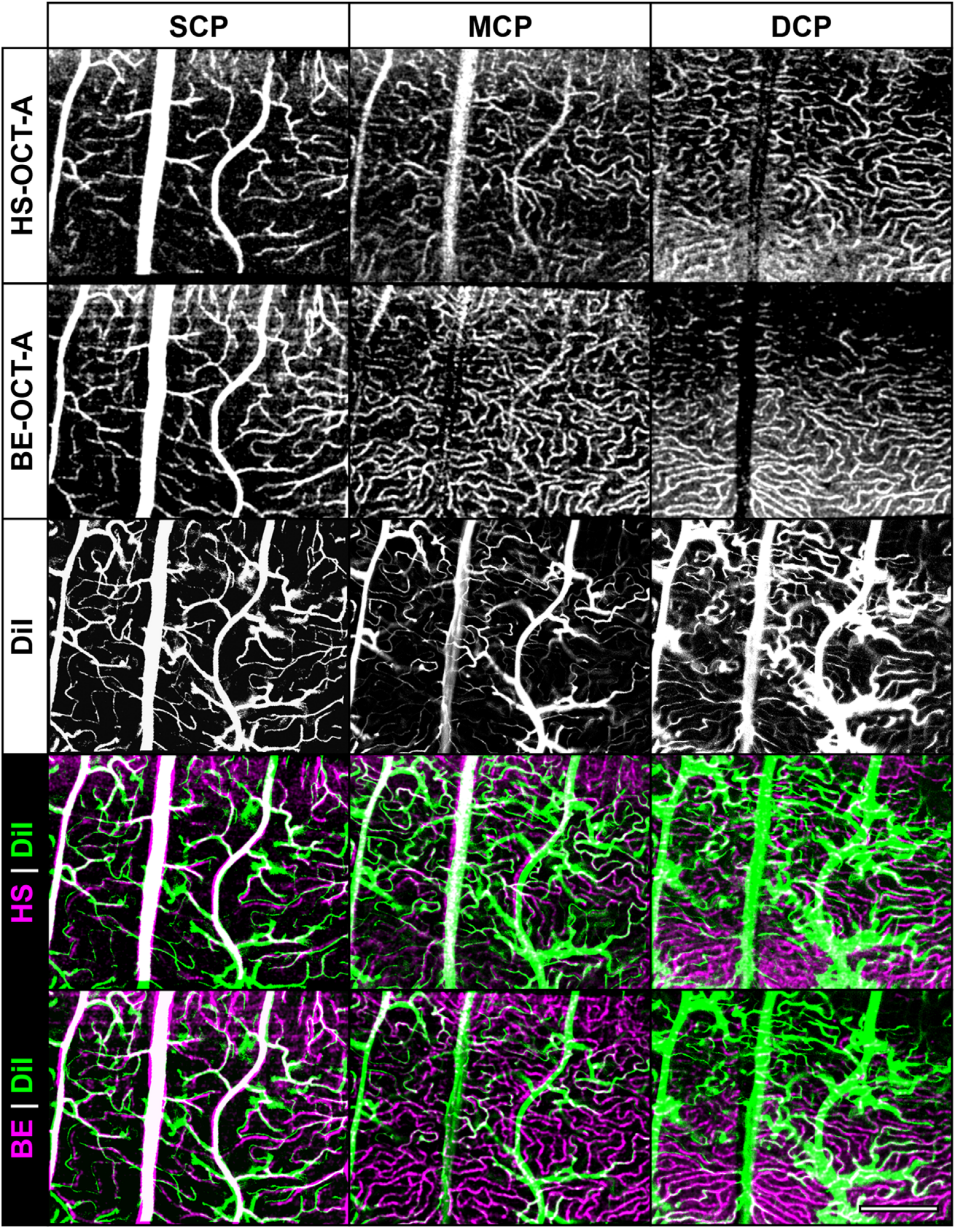
DiI-stained vasculature and OCT-A. Correspondence between in vivo OCT-A and ex vivo cardiac perfusion of DiI is generally good at all capillary plexuses, as overlap can be seen in many small capillaries (animal 164202: euthermic). The DiI staining in large and medium caliber vessels in the SCP is especially prominent and could not be optically sectioned from the MCP or DCP, resulting in large projection-like-artifacts with DiI-only signal. Additionally, DiI-only signal in the SCP can be seen indicating that the OCT-A sensitivity warrants improvement. However, DiI staining in capillaries was particularly weak in deeper layers, suggesting that other histological methods are warranted to obtain a more reliable frame of reference for OCT-A comparison. Scale bar: 250 µm.

After immunostaining with anti-α-SMA, the region previously imaged with OCT-A was visible in animal 187903 but not animal 164107. For animal 187903, vessel structure could be aligned with reasonable confidence; however, the SNR of these images was relatively poor (Supplementary Fig. S2) due to significant background signal, weak and disconnected signal originating from the capillaries, and prominent dissection artifacts associated with separating the neural retina from the sclera and ONH. Because DiI staining yielded considerably higher SNR, this approach was chosen for the remaining animals (*n* = 8). Correspondence between detected vasculature was assessed between the BE- and HS-OCT-A images acquired during euthermia and DiI images at each capillary plexus in animal 164202 (Fig. 8). DiI staining revealed vessels missed by both OCT-A devices, as well as vessels detected by both OCT-A devices that failed to stain with DiI. Due to projection artifacts^46^ and minor segmentation errors, several large- and medium-caliber vessels in the SCP were also seen in the MCP but not the DCP. The DiI signal, however, was extremely high in large- and medium-caliber vessels, could not be optically sectioned, and was therefore visible in all capillary plexuses.

The adjusted lateral scale of OCT-A images after rigid registration with DiI images was then compared to that predicted by our optical model (Appendix). With a 10° scan angle and 667 A-scans/B-scan, we estimated a pixel size of 1.5 µm given a retinal magnification factor (RMF) of 100 µm/degree,^28^ and this was not adjusted for axial length. After aligning the DiI micrographs with a calibrated lateral scale of 2.49 µm/pixel, the adjusted lateral scale of the OCT-A images was ∼1.84 ± 0.05 µm/pixel (mean ± SD, *n* = 6; Supplementary Fig. S3). The major difference between measured and predicted image scale (0.33 µm/pixel) may be attributable to some combination of histology artifacts, error in the assumed group refractive index, a species difference in RMF, and manual registration of the DiI images to the OCT-A images. Differences between animals may be attributable to small variations in axial length, refractive index, and error in manual registration of the DiI images to the OCT-A images.

## Discussion

We have presented a custom, high-speed OCT-A system, developed novel open-source software for an accessible commercial OCT system, demonstrated a translationally relevant OCT-A artifact in a natural model of transient ischemia, and provided a method for lateral scale calibration in ocular imaging. Below is a discussion on limitations of these products and findings, as well as suggested future directions of investigation.

### OCT-A Software

The acquisition and processing software for the HS-OCT-A was written with LabVIEW and MATLAB (both proprietary), which limits the accessibility of the software. Ideally, all software would be developed in a free, open-source environment to facilitate collaboration and advancement. Further, our acquisition software is highly specific to a spectral-domain point-scanning system and therefore cannot be immediately adapted to time-domain, swept-source, or full-field systems; a more generalized open-source framework for OCT systems would greatly benefit the community. Regardless, as SD-OCT-A represents a significant share of system designs, the current acquisition and processing software can be provided upon request to avoid a duplication of efforts.

Our B-scan registration algorithm (Appendix) thus far has only been tested in the 13-LGS but is likely to have sufficient performance in other animal models with minimal eye motion. There is currently no rejection of poorly correlated frames, and the limits of acceptable transformations were empirically derived for our set of image dimensions and range of eye motion seen in the 13-LGS. Before application to other species or systems, the registration parameters would thus require adjustment and calibration to image scale. The rationale for optimizing vertical shear instead of rotation was motivated by the maintained angle of vessel shadows during intentional offset pupil imaging.^47^ Applying a rotation instead of vertical shear would increase the magnitude and lateral extent of projection artifacts. Other registration techniques have shown excellent performance in humans in mitigating motion artifacts and increasing connectivity within vessels,^48^^–^^50^ and the acquisition software currently supports orthogonal fast scanning; however, this is not typically required in anesthetized animals, so a custom implementation of these algorithms was not pursued. It has also been shown that registering and averaging the *en face* angiograms improves SNR and vessel connectivity.^51^^,^^52^ A useful metric produced by future studies would be the optimal minimum number of volumes required to achieve a meaningful improvement in this species.

Currently, the capillary plexus segmentation is a costly, lossy, and subjective step in our OCT-A image processing workflow. Our platform for semiautomatic segmentation requires a separate offline step, wherein volumes are downsampled from 16- to 8-bits, jpeg compressed (which inherently rejects high-frequency information), and written to disk. The initial segmentation is not hardware accelerated and often requires manual adjustment of a segmented slab. Segmentation algorithms that attempt to identify the surface and inner plexiform/inner nuclear layer boundary have been demonstrated;^53^ future studies incorporating an automated segmentation step would increase the accessibility of this technique.

Finally, a few remaining software features affect the fidelity of vessel density measurements. Projection artifact resolution^54^ was not implemented in this study, so density estimates in the MCP and DCP are likely overestimated in regions with large overlying vessels. In the vessel segmentation algorithm, there are hard-coded parameters which limit its generalizability. Ideally, the sigma scale parameter would be inversely proportional to image scale; however, the nominal scale was equivalent for all images used in this study, and exhibited good performance, so an automatic adjustment of this parameter was not incorporated into the software at this time.

### Disambiguating Between Hypoperfusion and Capillary Dropout

When assessing the retinal vasculature, disambiguating between physical loss of blood vessels (dropout) and transient ischemia can have important diagnostic and prognostic implications. We found that, during torpor, patches of capillaries would be undetectable by OCT-A, reminiscent of apparent capillary dropout in diabetic retinopathy^55^ and retinal vein occlusion.^5^ This effect appeared more pronounced in the HS- than the BE-OCT-A device and, from the extremely low blood cell flux observed with AOSLO (Figs. 7A-D, Supplementary Video 1), it is likely that the effect is related to the interscan interval and repetition time of the OCT-A device. The scan protocols used with the HS-OCT-A device had a T_R_ for subsequent frames between 5.03 to 8.42 ms and a total BM-scan time between 10.1 to 40.2 ms, whereas the T_R_ and total BM-scan time for the BE-OCT-A device was approximately 14.7 ms and 58.8 ms, respectively. The camera of the HS-OCT-A device can support a range of A-scan rates between 80 to 250 kHz but enabling this feature would have required substantial hardware and software modifications. A technique referred to as variable interscan time analysis (VISTA),^7^^,^^15^ where interscan intervals are artificially extended by performing the decorrelation analysis on nonsequential B-scans within a BM-scan, has been demonstrated to increase the dynamic range of detectable flow velocities. Based on the square wave model generated from the slowest blood flow observed on AOSLO (Fig. 7F), VISTA is unlikely to restore visualization of all capillaries in the retina, as it would increase the effective interscan interval by a factor of ∼2 instead of ∼1000, but it may generally improve detection of slow flow. We recognize that this assertion is based on a single AOSLO video from a single animal, and that it is possible that the extremely low flux is not representative. Indeed, transient ischemic events have also been observed in nonconfocal AOSLO videos of the anesthetized mouse,^14^ and thus a more rigorous characterization of retinal blood cell flux between torpid and euthermic 13-LGS is warranted in future studies.

Alternative approaches beyond BM-scanning variants may also prove useful in addressing the issue of apparent capillary dropout disambiguation. One method to substantially increase the interscan interval while maintaining a practical workflow would be to perform repeated volume scanning.^56^ This method increases the likelihood that bulk eye motion would occur between repeated B-scans and would thus require a global registration prior to BM-scan registration, which is outside the scope of this study. As mentioned previously, multiple *en face* angiograms could be combined to simultaneously increase the effective interscan interval and improve SNR.^51^^,^^52^ The angiography algorithm may also affect the range of detectable blood cell flux. The algorithm we selected relies only on amplitude information; while fast and simple to implement, OCT-A contrast is then dependent on the passage of highly scattering blood cells. Phase-resolved OCT has been used to detect nanometer-scale changes in retinal cells,^57^^,^^58^ including retinal vasculature,^59^ and may be more robust to variable blood cell flux. Methods of tissue segmentation which do not rely on pixel-wise dynamic contrast in OCT have been demonstrated and represent another promising avenue for identifying nonperfused vessels. In another technique referred to as full-field swept-source OCT (FF-SS-OCT), an entire volume may be collected over the course of a single wavelength sweep (less than 1 ms).^60^ Scanning for a duration of five seconds with FF-SS-OCT to generate an OCT-A volume would be practical and may sufficiently detect even extremely slow flow in all capillaries.

### Correlative Histology

While the correlative histology performed in this study did not offer a reliable ground-truth metric for vessel density for comparison with in vivo OCT-A, the staining quality was sufficient to enable calibration of lateral scale, which is critical when comparing linear metrics between animals. For IHC images, the main limitation was relatively poor SNR. Improvement to SNR may be achieved using alternate target antigens such as von Willebrand factor or collagen-IV,^61^ as well as inclusion of an antigen retrieval step in the protocol. For DiI, the signal from large vessels was high and could not be optically sectioned well enough to obtain an isolated MCP or DCP image. Further, staining quality appeared to be inversely proportional to depth, suggesting either that the dye is less likely to be diverted to deeper layers through forced perfusion, or that the dye is more likely to be washed out of the deeper capillaries by the fixative. A protocol optimization examining the performance of multiple labeling methods including DiI, dextran, and lectin conjugated to a fluorescent probe,^12^ as well as factors such as volume, timing, fixation, mounting, and optical strategies is warranted and would provide a useful tool in validating OCT-A images. Regardless, even with poor staining in the deeper layers, the combined OCT-A/DiI approach offers an empirical method of determining image scale. The transverse scale of retinal images has been shown to be affected predominantly by axial length of the human eye.^62^^–^^64^ The magnitude of this effect in small eyes is poorly understood, and future studies are warranted to assess the correlation between image scale derived by optical models (incorporating noninvasive measurements of ocular biometry, such as keratometry,^65^ ultrasound,^66^ partial coherence interferometry,^67^ or whole-globe imaging with hyperparallel OCT^68^) and empirical measurements using correlative histology^69^ or application of exogenous agents with known size.^70^ Intraperitoneal or intravenous injections of DiI or lectin-FITC immediately preceding euthanasia may also be effective and require less technical skill, but this was not explored here.

## Conclusions

We have demonstrated the utility of a custom high-speed OCT-A system and performed a validation with a commercial OCT system. By leveraging the altered physiology during hibernation in the 13-LGS, we were able to perform within-animal assessments of altered vessel detection by OCT-A. These alterations were further investigated by AOSLO providing insights into the mechanism of OCT-A image artifacts. Through correlative histology, we provide a critical method for calibrating the lateral scale of ocular images in this species. This study provides a useful set of tools for analyzing aspects of retinal structure and function in an emerging animal model, as well as key insights into understanding the derivation of OCT-A images in the presence of altered physiology.

## Supplementary Material

Supplement 1

Supplement 2

Supplement 3

Supplement 4

## Appendix

### OCT and OCT-A Image Postprocessing

Dispersion compensation is critical to ensuring sufficient image quality for capillary imaging. Numerical dispersion compensation optimization is achieved by calling an external custom MATLAB function. When called, the input to the function is the most recent camera image used to display a B-scan to the user. The goal of dispersion compensation optimization is to identify coefficients of a phasor which minimizes group velocity dispersion and third-order dispersion, which in turn maximizes image sharpness in the axial dimension.^33^ We chose to optimize a sharpness metric, which is simply the sum of the OCT amplitude raised to the power of four. To identify the range of values this metric can take for a wide range of samples, we first induce empirically derived levels of extreme dispersion for each coefficient and measure the sharpness. The extreme coefficients are used to bound a nonlinear optimization using the MATLAB function *fmindbnd,* and the tolerance of the minimization is 10% of the difference between the sharpness values measured at positive and negative extremes. More robust methods of dispersion compensation have been proposed,^71^^,^^72^ but our chosen implementation is relatively simple, has sufficient performance, does not require separate acquisition of any images, and takes ∼0.40 seconds to complete (including the overhead associated with calling an external MATLAB script).

To improve visualization of layers in structural OCT images, an intensity transformation was performed. The same intensity transform was used for both the BE- and HS-OCT-A devices to facilitate ease of comparison. Both devices acquire data with 12-bits/pixel and store data in a uint16 format. Linear OCT intensities (initially 32-bit single precision after Fourier transformation), are transformed according to the formula:

$$\begin{array}{c} I^{'}\;=\;uint16\left(\log_{10}\left(\frac{I}{N}+1\right)\times S\right) \end{array}$$

Where *I* is the linear intensity, *N* is the FFT length (2048), *S* is an arbitrary intensity scaling factor (25_E_^3^), and *I*′ is the new display intensity.

To process BM-scans offline, a custom MATLAB workflow was developed to obtain structural scans as they were done online and register the frames within a BM-scan to mitigate decorrelation artifacts caused by eye motion. Each cluster of repeated B-scans was registered to an arbitrarily selected target frame (the middle frame in the sequence rounded up) using a custom registration algorithm. The registration algorithm is a nonlinear optimization function to adjust translation (dx, dy) and vertical shear (sh_y_) to minimize mean-squared error (MSE) between a source frame and the target frame. Similar to the dispersion compensation optimization, the range of MSE between images is calculated without any transformations applied and after applying hard-coded maximum displacement and shear levels (10 pixels and 0.05 δ_y_/δ_x_), then the termination tolerance of the minimization is 10% of this MSE range. Unlike the dispersion compensation optimization, dx, dy, and sh_y_ are adjusted simultaneously using the MATLAB function *fminsearch*. For the first registration in a cluster, an initial estimate of dx, dy, and sh_y_ of 0 is given, but performance is improved by using the output of this registration as the initial estimate for subsequent registrations. We have assessed the magnitude of tolerance for the transformation coefficients and find, for our set of image dimensions, that a tolerance of 1_E_^−6^ pixels results in sufficient mitigation of decorrelation artifacts due to poor registration but comes at a cost of processing time (Supplementary Fig. S1). While artifacts parallel to the fast-scan axis may be mitigated by filtering, this increases the risk of erasing or disconnecting capillary segments. Registered frame clusters are then averaged to produce a high SNR structural volume. The same registered frames are subjected to a custom implementation of full-spectrum amplitude decorrelation angiography (FSADA).^34^ The amplitude mask threshold was selected to be the mean + 1 SD of all intensities in the registered and averaged structural B-scan (Fig. 2). Structural and FSADA volumes were output as jpeg compressed .avi files for use with an existing segmentation platform, discussed below.

The registered/averaged structural volumes were then segmented using a custom implementation^73^^,^^74^ of the graph theory and dynamic programming algorithm^53^ (designed for humans, but has been previously demonstrated in the 13-LGS^16^). The inner limiting membrane (ILM) and nerve fiber layer/ganglion cell layer segmentations were then applied to the angiographic volume and manually shifted deeper into the SCP (∼35 µm from the ILM) using the *en face* mean intensity projection (computed in real-time) for visual guidance. At this point, the better segmentation was subjectively determined by examining the continuity of vasculature in the *en face* projection and manually corrected as needed to improve continuity and avoid segmenting the wrong layer. This correction was done by adjusting control points of a custom contour with cubic interpolation between control points on all B-scans in the volume using the decorrelation signal in the B-scan for visual guidance. The SCP segmentation was then copied and manually shifted deeper into the MCP and DCP and individually corrected as above (∼75 and 120 µm from the ILM, respectively). The DCP, in particular, required substantial correction close to the ONH as it would converge in depth with the MCP. The thickness of each mean intensity projection ranged between ∼14 to 30 µm (centered about the segmentation) in an attempt to compensate for small segmentation errors without including too much background signal, which degrades the SNR of the mean intensity projection. Angiograms were then imported to Photoshop, layers from the same scan were linked, and angiograms from different scans (different timepoints/retinal locations) were manually aligned using vessel branch patterns from all layers to compare images from different devices or thermic states. When comparing images between animals, the largest overlapping ROI between devices and/or timepoints within an animal was first found, then truncated to the smallest of these ROIs between animals. The size of each ROI was calibrated using a lateral scale determined by correlative histology (see *Cardiac Perfusion Labeling of Retinal Vasculature*).

### 13-LGS Eye Model and Resolution Calculations

From a previous report of the dimensions of mouse retinal capillaries imaged with AOSLO, the minimum diameter measured was 3.2 µm with a mean ± SD of 4.0 ± 0.7 µm, with ∼95% of observations ≥ 3.5 µm.^14^ With this in mind, we targeted a pixel size of 1.5 to 1.75 µm for the HS-OCT-A to ensure adequate sampling. Using a retinal magnification factor of 100 µm/degree of visual space (based on measurements in the European ground squirrel)^28^ galvanometric scan angles were converted to linear areas to obtain an approximate lateral scale. With an estimated 1/e^2^ beam diameter at the cornea (*d*) of 2.1 mm (Thorlabs, CFS11-850-APC), posterior nodal distance of ∼5 mm (*f*), a center wavelength (λ) of 850 nm, and a group refractive index of 1.38 (*n_tissue_*), we computed a lateral resolution (δ_*x*,*y*_; half Airy disk diameter according to the Rayleigh criterion) of 1.85 µm according to the formula:

$$\begin{array}{c} \delta_{x,y}\;=\;\frac{1.22\lambda f}{d}\times \frac{n_{air}}{n_{tissue}} \end{array}$$

This is likely somewhat distorted by ocular aberrations, although the fully dilated 13-LGS pupil diameter is ∼4.5 mm, and aberrations as a function of pupil size have not been measured yet in this species. Regardless, the HS-OCT-A images are likely slightly oversampled, and speed could be improved in future studies. An axial resolution of 1.40 µm was calculated according to the formula:^24^

$$\begin{array}{c} \delta_{z}\;=\;\frac{2\ln\left(2\right)}{\pi }\times \frac{\lambda_{0}^{2}}{\Delta \lambda }\times \frac{n_{air}}{n_{tissue}} \end{array}$$

Where the central wavelength (λ_0_) was 850 nm, and the bandwidth (Δλ) was 165 nm.

### Maximum Permissible Exposure (MPE)

For the HS-OCT-A, measured laser power at the cornea was ∼3.26 mW at λ_0_: 850 nm. Exposure was first modeled as a uniformly irradiated square with the most conservative parameters (smallest FOV: 7° × 7°, all power at shortest wavelength: λ_0_ − Δλ/2 = 767.5 nm, rather than a spectrally weighted function favoring the NIR-range), as was suggested in section 7.E. of Delori et al.^20^ using updated parameters from ANSI Z80.36-2016.^21^ Exposure time for a single volume was 2.20 seconds (1.13 ms/B-scan was included as the SLD was not disabled during flyback). The calculated radiant exposure at the 13-LGS retina was 1.86J/cm^2^/pulse, which exceeds the photothermal radiant exposure limit of 14.93J/cm^2^ after eight pulses. As no BM-scan protocol included more than eight B-scans, this power level was considered relatively safe during scan acquisition. While aligning the pupil, however, only line scans are used, so the exposure was then calculated using a spot with a minimum retinal dimension of 30 µm.^21^ The calculated retinal radiant exposure was 217mJ/cm^2^/pulse, which exceeds the photothermal radiant exposure limit of 2.4J/cm^2^ after ∼52 seconds. This is typically a sufficient duration for aligning the pupil, but a few hardware/software changes may dramatically decrease the laser hazard for future studies. These include disabling the SLD during flyback, automatically alternating between orthogonal line scans while aiming, and exposing at the display rate (as opposed to the maximum rate) while aiming. Finally, the radiant exposure limits are defined using data derived from different species,^75^ the optical model for the 13-LGS is lacking, as is our understanding of how heat dissipation may be impacted by the reduced blood flow during torpor.
