# Supplementary material for: Optical Coherence Tomography Angiography in the Thirteen-Lined Ground Squirrel
Source: Transl Vis Sci Technol. 2021 Jul 7;10(8):5. doi: 10.1167/tvst.10.8.5 (PMC8267221; doi:10.1167/tvst.10.8.5)
Supplement: Supplement 1 [file tvst-10-8-5_s001.pdf]

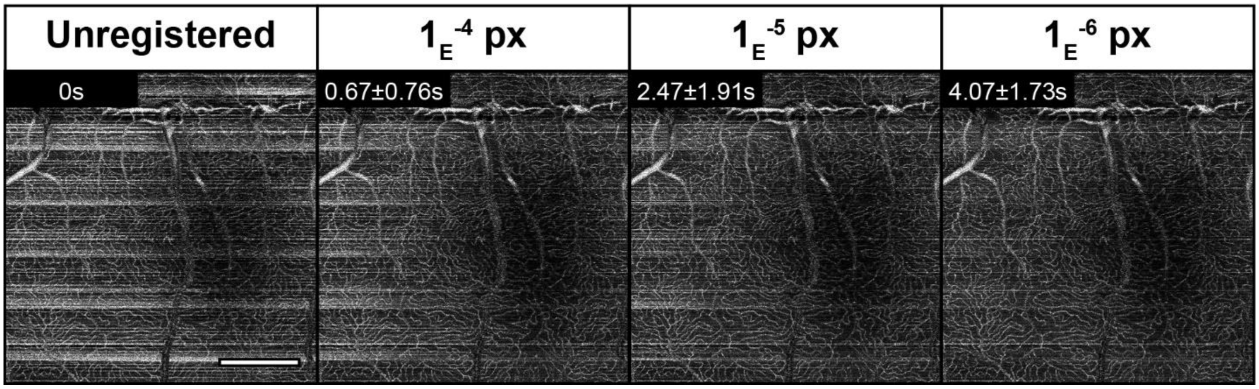

Supplementary Figure 1 – Registration performance as a function of tolerance. Shown is an image of the MCP acquired with the BE-OCT-A device from animal 187903 in the winter-active state. The termination tolerance of the registration parameters (dx, dy, and sh_y) was varied to assess the effect of optimization precision on decorrelation artefacts and processing time. The segmentation was performed on the volume registered with 1_E^{-4}-pixel tolerance and applied to the other volumes. The mitigation decorrelation artefacts due to eye-motion with 1_E^{-6}-pixel tolerance was determined to be sufficient for analysis and only results in ~6x speed decrease relative to 1_E^{-4} pixels (mean ± SD registration computation time per frame is given for each volume). Scale bar: 500µm.
