# Supplementary material for: Optical Coherence Tomography Angiography in the Thirteen-Lined Ground Squirrel
Source: Transl Vis Sci Technol. 2021 Jul 7;10(8):5. doi: 10.1167/tvst.10.8.5 (PMC8267221; doi:10.1167/tvst.10.8.5)
Supplement: Supplement 2 [file tvst-10-8-5_s002.pdf]

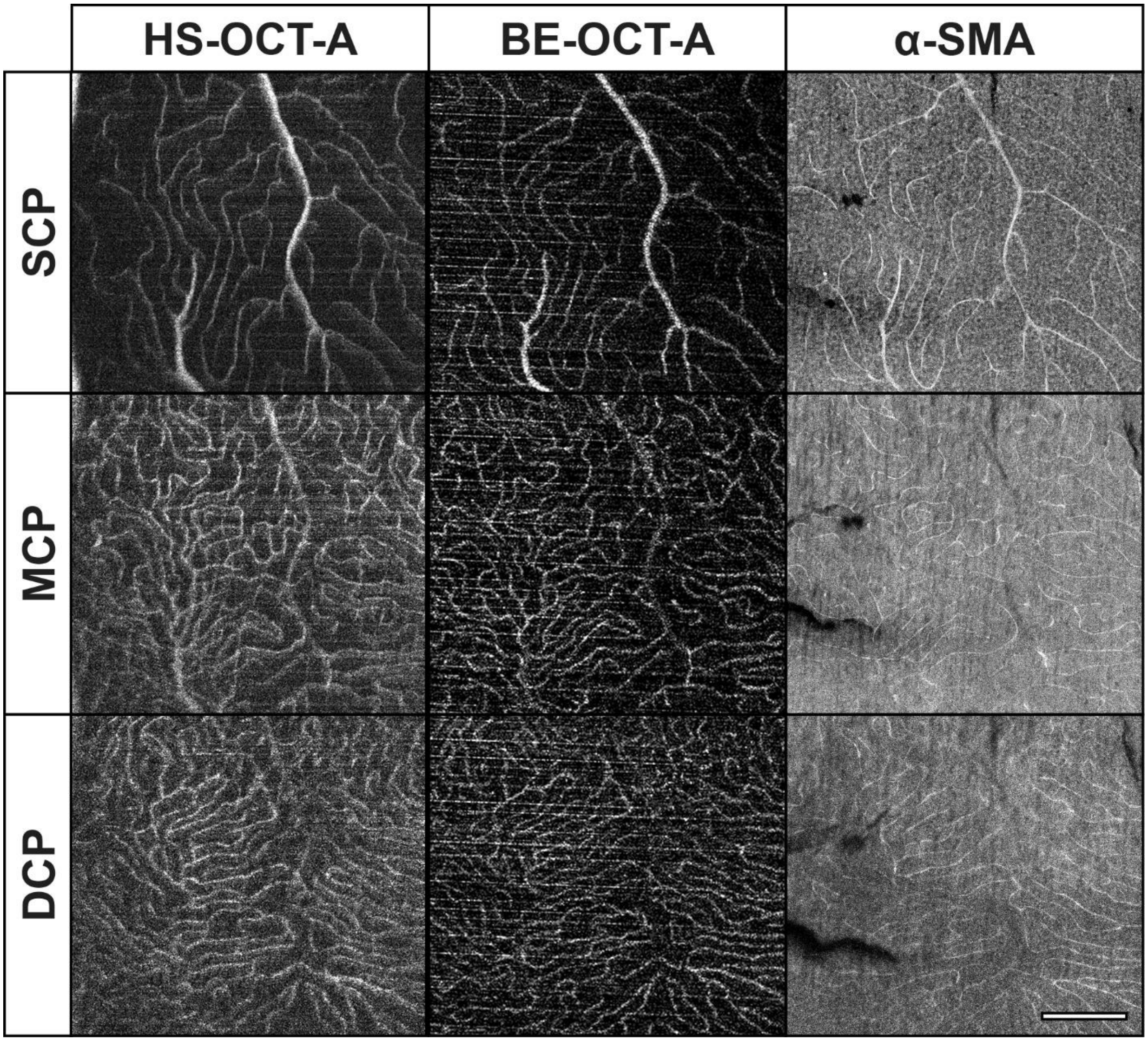

Supplementary Figure 2 – Immunohistochemical labeling of the retinal vasculature and comparison with *in vivo* OCT-A. In animal 187903, many of the same vessels may be seen in all three modalities enabling lateral scale calibration; however, the SNR of the immunolabeling with anti-α-smooth muscle actin (α-SMA) should be improved before a quantitative assessment of morphologic correlation is pursued. Scale bar: 200µm.
