# Supplementary material for: Optical Coherence Tomography Angiography in the Thirteen-Lined Ground Squirrel
Source: Transl Vis Sci Technol. 2021 Jul 7;10(8):5. doi: 10.1167/tvst.10.8.5 (PMC8267221; doi:10.1167/tvst.10.8.5)
Supplement: Supplement 3 [file tvst-10-8-5_s003.pdf]

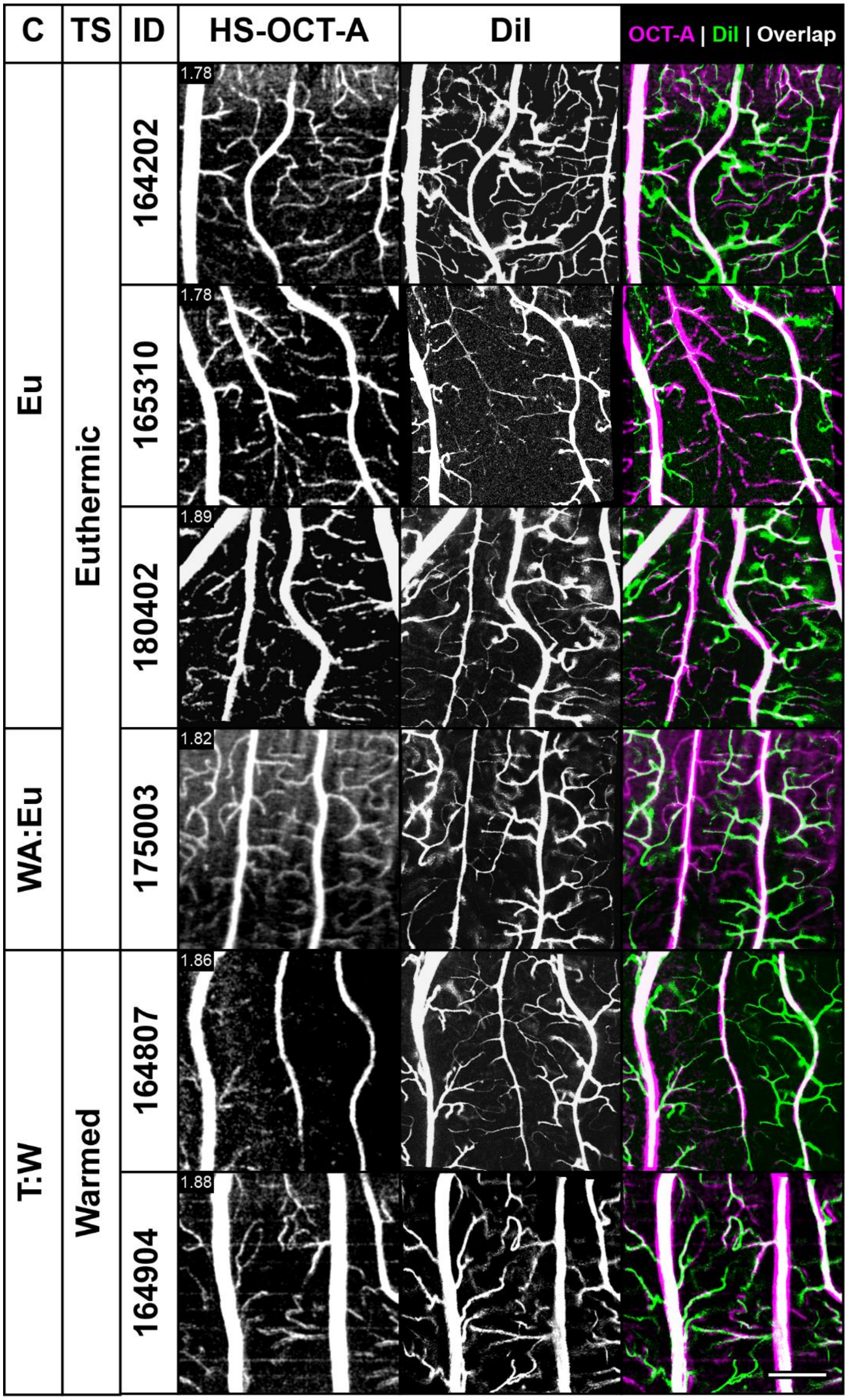

Supplementary Figure 3 – Lateral scale calibration with DiI stained retinal vasculature. DiI micrographs were aligned to the superior capillary plexus (SCP) image acquired with the HS-OCT-A. The cohort (C), thermic state (TS), and ID for each animal are indicated on the left. The known lateral scale of each DiI image was used to adjust the nominal lateral scale of the OCT-A image (indicated in the top left corner of each OCT-A image; units: µm/px). All images shown were adjusted to have equivalent scale in this figure. Scale bar: 200µm.
